# Allergic shock caused by ingestion of cooked jellyfish: A case report

**DOI:** 10.1097/MD.0000000000007962

**Published:** 2017-09-22

**Authors:** Zhixing Li, Xungang Tan, Botao Yu, Renliang Zhao

**Affiliations:** a Qingdao University Medical College, Qingdao; b Key Laboratory of Experimental Marine Biology, Institute of Oceanology, Chinese Academy of Sciences, Qingdao, Shandong; c Emergency Department, The Affiliated Central Hospital of Qingdao University, Qingdao; d Department of Neurology, The Affiliated Hospital of Qingdao University, Qingdao; e Laboratory for Marine Biology and Biotechnology, Qingdao National Laboratory for Marine Science and Technology, Qingdao, China.

**Keywords:** allergic shock, clinical allergy, food allergy, jellyfish, urticaria

## Abstract

**Rationale:**

Although anaphylaxis caused by jellyfish stings is common in coastal areas, an allergic shock caused by cooked jellyfish ingestion has never been reported in China. In this paper, we report a case of allergic shock being caused by ingestion of cooked salt-preserved jellyfish shortly after being stung by a live jellyfish.

**Patient concerns:**

A 26-year-old Chinese man presented with dizziness, pruritus, dyspnea, hypotension, and tachycardia after eating cooked salted jellyfish. The patient had been stung twice by jellyfish half a year ago.

**Diagnoses:**

Allergic shock caused by ingestion of cooked jellyfish.

**Interventions:**

The patient was treated with phenergan (25 mg, intramuscular injection), 250 mL normal saline (NS) and 10 mg dexamethasone (intravenous drip), 500 mL NS and 0.4 g cimetidine (intravenous drip), and 500 mL NS for rapid fluid infusion (intravenous drip).

**Outcomes:**

After the treatment, the main clinical symptoms of the patient improved quickly. Five days later, the patient's urticaria had dissipated.

**Lessons:**

A history of jellyfish contact or sting might be an important allergic factor for individuals who consume any kind of jellyfish.

## 1 Introduction

In recent years, an increasing number of cases of jellyfish stings have been reported on coastal beaches throughout the world. Jellyfish are marine invertebrates belonging to the class Scyphozoa of the phylum Cnidaria. The body consists of tentacles covered with cnidocytes and venom.^[1,2]^ Jellyfish stings may induce immediate or delayed toxic reactions.^[3–5]^ Upon contact with the tentacles, the cnidocytes may penetrate the skin and inject the venom, causing nematocyst dermatitis, anaphylactoid reaction, shock, and even death.^[1–3]^ Salt-preserved jellyfish is a common food in China, especially in coastal areas, and there have been some reports of severe systemic anaphylaxis induced by ingestion of jellyfish months after being stung by a jellyfish elsewhere in Asia.^[6,7]^

## 2 Case presentation

A 26-year-old Chinese male developed symptoms of erythema, pruritus, and palpitation half an hour after eating cooked salt-preserved jellyfish. The symptoms worsened shortly after the patient arrived at the hospital. He exhibited dizziness, dyspnea, and tachycardia. The patient was otherwise healthy, had no history of allergies to drugs or other substances, and had been stung severely by jellyfish approximately 6 months prior. The patient had first eaten some cooked beef and vegetables and showed no symptoms. Within 15 minutes of eating the jellyfish, the patient began to develop symptoms. No beverages or alcohol were consumed during this period.

**Figure 1:**
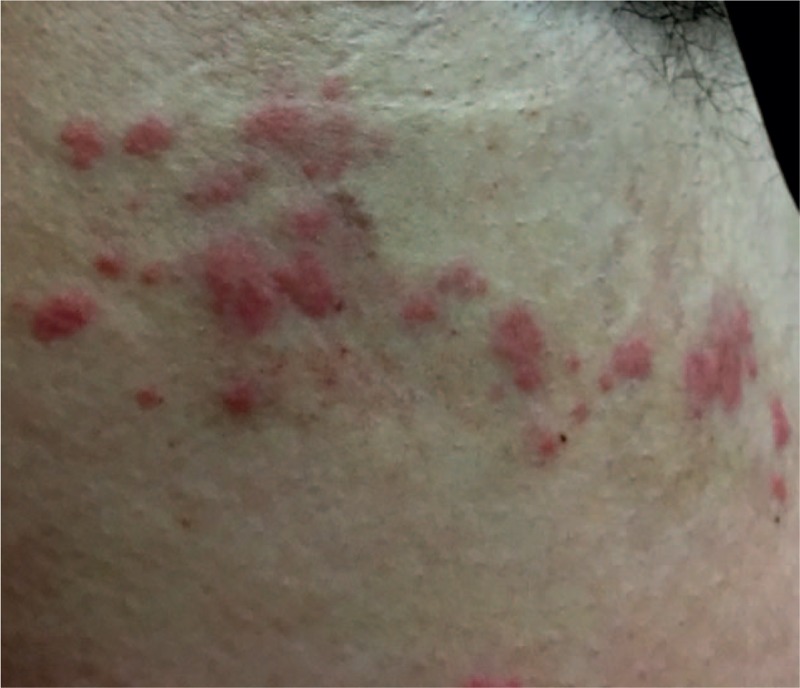
Clinical appearance of the patient. There are obvious sting marks and pigmentation around the allergic region on the right side of the trunk, near the armpit.

The physical examination showed that the patient was conscious, with hypotension (86/50 mm Hg), hyperpnea (Kussmaul respiration and hyperventilation), tachycardia (137 beats per minute [BPM]), and a regular heart rhythm. He also had cheek and pharynx swelling, flushing of the face, neck, and chest, limb stiffness, and upper limb and armpit urticaria (Fig. 1). An electrocardiogram showed sinus tachycardia and no other abnormalities. The preliminary diagnosis was allergic shock.

The patient was treated with phenergan (25 mg, intramuscular injection), 250 mL normal saline (NS) and 10 mg dexamethasone (intravenous drip), 500 mL NS and 0.4 g cimetidine (intravenous drip), and 500 mL NS for rapid fluid infusion (intravenous drip). He was kept for observation in the emergency department, and his blood pressure was continually measured.

After 1 hour, the main clinical symptoms of the patient had improved. Both blood pressure and heart rate had returned to normal (130/86 mm Hg and 86 BPM, respectively). Furthermore, the patient displayed no palpitation, dizziness, limb stiffness, or other symptoms. After treatment and fluid infusion, the patient was given an oral antiallergic agent (loratadine tablets, 10 mg/d for 1 wk) and educated regarding a safe diet. Five days later, the patient's urticaria had dissipated.

The patient had provided the consent to publish the case report, and the consent procedure was approved by the Ethics Committee of the Affiliated Hospital of Qingdao University.

## 3 Discussion

Jellyfish venom contains a high concentration of medusocongestin, which mainly consists of proteinous toxins, polypeptides, enzymes, histamine, 5-hydroxytryptamine, and other bioactive molecules.^[1,8]^ When these toxins enter the human body, they may cause extensive angiotelectasis, leading to increased vascular permeability, and subsequently, a rapidly decreased circulatory blood volume. This may lead to refractory hypotension, acute cardiac failure, and even death.^[1,9]^ In this case, it is likely the allergic shock was caused by the ingestion of salted jellyfish, as previously reported.^[6,7,10]^ However, in this case the salted jellyfish was cooked before being eaten, and there are also other interesting points in this case worthy of attention.

The first one is the patient's history of being stung by jellyfish. The patient had, on previous occasions, been stung by jellyfish and gone on to eat salt-preserved jellyfish, without any anaphylaxis. The time interval was similar; however, the difference in this instance was the repetitive nature of the jellyfish sting. The patient was stung by jellyfish twice 6 months before admission. Local cutaneous reactions were severe the first time, and slight the second time. The second point to consider is the position of the patient's urticaria after jellyfish ingestion: it was localized to the same region where the patient had been stung 6 months prior. This suggests that the urticaria was not caused by a simple delayed toxic reaction, as in the other reports, but rather from a systemic allergic reaction.^[1,5]^ We speculated that this might have been a consequence of antibody accumulation at the site. Salt-preserved jellyfish might contain related antigens that could trigger an antigen–antibody reaction upon ingestion, manifesting in these positions and further causing systemic symptoms. The third point is the difference in reaction among different individuals. Three relatives of the patient were also stung severely by jellyfish 6 months prior, and ingested the same salt-preserved jellyfish at the same time. However, they did not experience any anaphylaxis or discomfort. The differences between our patient and his relatives, such as in age, sex, alcohol intake, and other factors, might affect the allergic response to jellyfish toxins, but more study is needed to clarify the associations. The final point is the treatment that was administered after the jellyfish sting. The patient was treated with a customized antidote for jellyfish venom designed by the Institute of Oceanology of the Chinese Academy of Sciences.^[11]^

There are few reports of anaphylaxis after jellyfish ingestion; however, there have been 3 recent responses of anaphylaxis after jellyfish ingestion have been reported in patients with or without a history of frequent jellyfish stings.^[6,7,10]^ In these cases, the allergic reaction was induced by salted jellyfish, which contains an antigen that could induce allergic responses. However, in our case the jellyfish was not only salt-preserved, but also cooked, and as such antigen availability would not be comparable to that of uncooked jellyfish. One possible reason is that short peptides in cooked jellyfish, which are the degraded fragments of the proteins found in living and salted jellyfish, might be the antigens. A previous study found that the injection of T-cell reactive peptides could safely improve human allergic responses to cat dander.^[12]^ Also, another possibility is that bioactive substances, which are produced by jellyfish, could be absorbed in the digestive tract. These substances might be important in the process of allergic reactions in humans. Although numerous studies have demonstrated that allergen-specific immunoglobulin E (IgE) and some food antigens can lead to anaphylaxis,^[13]^ there are only 2 reports of the production of jellyfish-specific IgE or immunoglobulin G (IgG) antibodies in response to jellyfish stings.^[4,14]^ Therefore, the underlying mechanisms linking the allergic reaction to jellyfish consumption and jellyfish stings are unclear.

In conclusion, people should be cautious of the possibility of allergic reactions to jellyfish after ingestion. A history of jellyfish contact or sting should be taken into serious consideration when eating any kind of jellyfish, within at least 1 year after being stung by a jellyfish.
